# The Foreign Journals: Medical Jurisprudence and Toxicology

**Published:** 1842-01

**Authors:** 


### MEDICAL JURISPRUDENCE AND TOXICOLOGY.

*Case of Alleged Childmurder.* By Dr. GRAFF, of Darmstadt.

E. B. of F——, æt. 24, the female charged with the crime, was suspected to be pregnant, by her parents and neighbours; but she denied the fact, although well aware of her condition. On the night of the 10th November, after having taken violent exercise in dancing, she retired to rest but suffered from severe pain. The following facts were obtained from the confession which she subsequently made to the civil authorities:

About three o'clock in the morning she rose from her bed, and went to a neighbouring stable, where she was suddenly delivered, the child falling from her on the floor. At this time *she heard the child cry.* Fearing that this might create an alarm, she seized a knife, and cut at the body of the child, but where she wounded it, owing to the darkness, she could not say. Immediately after she had thus used the knife, the crying of the child ceased. The placenta came away about a quarter of an hour afterwards, and this with the body she buried in a heap of dung.

This woman was examined on the 23d November, and it was found that she had been recently delivered. She made a full confession, owing to which the body of the child was discovered, and on the following day it underwent a medico-legal inspection. The body, which was that of a female child, was about eighteen inches long. The skin had begun to separate by putrefaction. The bones of the cranium were flattened without presenting any mark of fracture; and the brain had been forced out. The face presented marks of violence, but the openings of the mouth and nostrils were free. The umbilical cord had been torn through at about two inches from the abdomen. The right forearm had been separated at the elbow-joint from the upper arm. The viscera of the chest and abdomen were in a state of putrefaction.

The lungs over their whole surface were covered with dark-looking vesicles containing air; they were expanded, and filled up the cavity of the chest, except in the fore-part, which was occupied by the thymus gland, also in a state of putrefaction. These organs when placed together on temperate water floated. The lungs when placed by themselves on water floated above the surface.

On cutting into them for about one fifth of their substance from the surface, a crepitating sound was perceived. This portion was of a dark colour, light, and spongy. The internal substance of the organs was brown and firm; and on cutting into it no crepitation was perceived, nor did any blood escape. The pieces into which this inner portion of lung was divided readily sunk in water. The heart was black from putrefaction, and there was some decomposed blood in its cavities. The foramen ovale was open.

Among other appearances it was observed that the epiglottis was closed.

In his report, the examiner gave it as his opinion that this child *had not breathed.*

Although putrefaction had so far advanced as to render any evidence from the application of the hydrostatic test doubtful, yet he thought from the firmness, colour, and great specific gravity of the inner portion of the lungs, which was not putrefied, as well as from the total absence of air-cells, that there was sufficient ground to infer that respiration had not been performed. This opinion was corroborated in his view by the facts that the epiglottis was closed, and the foramen ovale in the septum of the auricles was open.

Another question put to him was, whether the obstruction to respiration had proceeded from accidental or intentional causes, which he answered by stating that the child, in his opinion, had come into the world dead.

The flattening of the skull and extrusion of the brain, he ascribed to some person having accidentally trodden over the dung-heap where the child was buried, some time after its body had advanced into a state of putrefaction. The separation of the right fore-arm, evidently from tearing and not from wounding, had probably taken place under similar circumstances.

The air-vesicles on the surface of the lungs, as well as the inflation of a portion of their substance, were an evident result of putrefaction. The same may be said of the buoyancy of the heart and thymus when placed on water. The air-vesicles in these organs, but especially those in the lungs, seemed to keep the viscera on the surface of water. The death of the child, within the body of the mother, was attributed to her having, during the latter part of her pregnancy, worn a tight bandage round the abdomen, as also to her having over-exerted herself in dancing the night before her delivery.

The medical opinion thus expressed being in direct opposition to the confession made by the prisoner, the case was referred by the legal authorities to one of the Hessian medical colleges. The members were directed to reconcile the facts if possible with the confession, and to give a judgment on the medical report. They examined the questions seriatim, and first that relative to respiration after birth.

After examining the grounds on which the medical opinion was expressed, and making due allowance for the advanced state of putrefaction in the body, owing to its having been buried in the dung-heap, they came to the conclusion that there was a *certain degree (!)* of probability, that the child had breathed. They complain that the heart and thymus were not placed on water separately; since their buoyancy may, under the circumstances, have been due, not to their being in a putrefied state, but their receiving support from the air in the lungs. They contend that the most putrefied lungs which have not respired sink in water, and that the air of putrefaction, contained only in a thin layer on the surface of the lungs, would rarely, if ever, suffice to give buoyancy to the lungs, heart, and thymus at the same time. If the examiner supposed that the air in the thoracic viscera was derived from putrefaction, he ought to have used compression, and then again tried the hydrostatic test. This would at once have settled whether the air was due to putrefaction or not.

In looking at the facts with the most lenient eye, and admitting that putrefaction had advanced so far in this case as to destroy all the usual marks of respiration, it must at the same time be allowed that the conclusion drawn from the colour and consistency of the substance of the lungs was unjustifiable, and decidedly erroneous.

The sinking of the lungs either wholly or in part, they contend, is no proof that the child has not lived and breathed. Many cases are recorded which establish the truth of this statement, and they refer to Henke for facts, which show that children whose lungs have sunk in water have been known to live, breathe, and cry. They consider that the state of the epiglottis and foramen ovale furnishes no evidence on which reliance can be placed. The open state of the mouth and nares is quite compatible with the child having been wilfully suffocated, or its respiration intentionally suppressed; and, on the whole, they contend that the opinion first expressed, that this child had been born dead, was false and untenable.

They object to the explanation given of the marks of violent injury on the body, and think that they might rather be accounted for by reference to the weapon employed by the mother. The conduct of the inspector is condemned, because he was made aware of the confessions of the prisoner before he drew up his report, and he should therefore either have reconciled the medical facts with the confessions, or have clearly shown in what respect the confessions were inconsistent with them. They close their judgment by the remark, that there was no ground whatever for asserting that any of the facts of the case were in opposition to the confessions made by the prisoner.

[REMARKS.—We have selected this from many other cases of infanticide reported in the same journal, owing to its presenting some points which are deserving the attention of the English medical jurist. We assume that the confession made by the female was true: there seems to have been no reason to suppose that she wilfully misrepresented the circumstances, and we shall now compare these with the medical inferences.

The practitioner who examined the body came to the conclusion that the child was born dead and had not performed the act of respiration. We agree with him that the buoyancy of the lungs was due to putrefaction, and it appears to us that the reasons adduced against this conclusion by the medical college are wholly unsatisfactory. The inference to which they come, that the child had respired, appears to be an ex post facto conclusion, not warranted by any of the appearances met with on inspection, and apparently grounded only on the confession of the female that she had heard the child cry for about a minute after delivery. Had this not been known to them, we doubt whether they could have conscientiously affirmed that there was a single appearance indicative of respiration. Much was omitted, it is true, by the first examiner, but in this fact we have rather a want of evidence than a reason for supporting an opinion by negative assumptions.

We agree in their condemnation of the report, but not for the reasons which they assign. The case was beyond the reach of medical evidence, owing to the body of the child being in such an advanced state of putrefaction; there was no ground for asserting that the child was born dead, nor was there any proof of its having been born living. The facts of the case were certainly not in opposition to the confession made by the mother; for it is possible that she may have heard the child cry, without its being necessary that its lungs should float in water. The Medical College are indignant at the inference that the air-vesicles seen in the lungs arose from putrefaction only; yet they immediately afterwards admit, on unquestionable authority, that children whose lungs sunk in water have been known to live, breathe, and cry. Hence then the discovery of air in the organs was not, on their own view, necessary to account for the feeble cry which the mother heard. The error committed by the physician who examined the body was, that he took the sinking of the central portion of the lungs as direct evidence of still-birth, the floating of the superficial portions being satisfactorily accounted for by putrefaction: but in this he only followed what is a general rule of the profession.

Notwithstanding that it is now well known that new-born children live and breathe for many hours and even days, and yet their lungs wholly or partially sunk in water, it is the custom at most coroner's inquests to take this as direct evidence of still-birth. The impropriety of this practice is clearly shown by the case reported; for here the confession of the mother proved, that this child must have been born living, while the medical rule usually followed would have led to the inference that it was born dead. If females charged with the crime in this country were more frequently to make candid confessions, we should probably often find these in direct opposition to the medical opinions given before a coroner. There can be no doubt that many children are pronounced still-born on this erroneous principle, and the case is at once disposed of. No further enquiry is made, and the murderess probably owes her escape to the prevalence of this false medical doctrine. We may in some measure satisfy ourselves on this point, by comparing the proportional number of so-called still-births among children which are the subjects of coroners' inquests with the ascertained proportion of still-births among illegitimate children.

The course of conduct for a medical witness to pursue in such a case, when the body of a child is not so putrefied as to render an examination useless, is to content himself by saying, that there is a want of evidence of life after birth, and not to affirm from the sinking of the lungs that the child must have been born dead. This condition is certainly a presumption in favour of still-birth, but nothing more; and if in neglect of this rule we state that the child could not have survived birth, we must be prepared to have our opinion contradicted by the confession of the prisoner, as by circumstances of the existence of which we were not aware.

But it may be asked, can it be admitted that a child in whose lungs no air is found should have had the power to cry? We think the present case satisfactory on this point, although others are not wanting which show that children whose lungs sunk in water have been heard to cry; in these cases the cry appears to have been always feeble, and the air which produced the sound probably came from the larger divisions of the bronchi, and not from the substance of the lungs. There is a curious case reported in the present number of this Journal, (p. 243,) which shows the possibility of this, even in the adult.]

*Henke's Zeitschrift.* 27 *Ergänzungeheft.* 1840.

